# 

**Published:** 1960-03

**Authors:** 


### *Contents*

| | | |
|---|---|---|
| DOMESTIC HAZARDS WITH NEUROLOGICAL SIGNIFICANCE | *A. M. G. Campbell* | 23 |
| IGNAZ PHILIPP SEMMELWEIS AND HIS TIMES | *Frederick Kohn* | 29 |
| "RENAL COMPLICATIONS OF DIABETES MELLITUS" | *Professor T. F. Hewer* | 37 |
| NOTES AND NEWS | | 41 |
| EXAMINATION RESULTS | | 41 |
| APPOINTMENTS | | 41 |

